# Ablative stereotactic MR-guided radiotherapy for >10 cm solid pseudopapillary tumor of the pancreas with liver oligometastases: a case report and literature review

**DOI:** 10.3389/fonc.2026.1808600

**Published:** 2026-05-04

**Authors:** Robert A. Herrera, Nikolai Strusberg-Fernandez, Eyub Y. Akdemir, Fernando De Zarraga, Horacio Asbun, James McCulloch, Nema Bassiri, Michael D. Chuong

**Affiliations:** 1 Department of Radiation Oncology, Miami Cancer Institute, Miami, FL, United States; 2 Department of Medical Oncology, Miami Cancer Institute, Miami, FL, United States; 3 Department of Surgical Oncology, Miami Cancer Institute, Miami, FL, United States

**Keywords:** ablative radiotherapy, liver metastases, MR-guided radiotherapy (MRgRT), oligometastatic disease, pancreatic cancer, rare tumors, solid pseudopapillary tumor (SPT)

## Abstract

**Background:**

Solid pseudopapillary tumors (SPTs) of the pancreas are rare, low-grade malignancies that generally have favorable outcomes when diagnosed at an early stage and can be resected. However, treatment options for patients with unresectable or metastatic disease are limited, and the role of radiotherapy (RT) is not well defined, especially for lesions near dose-limiting organs at risk (OARs). Advances in magnetic resonance–guided radiotherapy (MRgRT) can facilitate safe dose escalation for anatomically unfavorable tumors that may improve clinical outcomes. We report the use of dose-escalated MRgRT for >10 cm pancreatic SPT with liver oligometastases.

**Case summary:**

A 63-year-old female was incidentally found to have a 7-cm mass in the pancreatic body and three hepatic lesions with biopsy confirmation of SPT. Surgery was attempted after 3 months of chemotherapy but aborted intraoperatively due to extensive vascular involvement. She received four cycles of FOLFOX followed by definitive RT to the primary tumor that had enlarged to 10 cm. Given the tumor’s size and extensive abutment of the stomach and bowel, she was treated on a 0.35-Tesla MR-Linac using a dose-painted, simultaneous integrated boost with 50.4 Gy in 28 fractions prescribed to the primary tumor and 75.6 Gy in 28 fractions prescribed to a central tumor volume. Concurrent chemotherapy was not used. One month later, two of the three liver metastases were treated with MRgRT to 40 Gy in five fractions. One sub-centimeter liver metastasis was observed due to concerns about cumulative liver dose, although due to eventual slight progression, it was treated 19 months later with the MR-Linac to 35 Gy in one fraction. All RT courses were well tolerated with no grade ≥2 toxicity. She is alive, asymptomatic, and without evidence of progression nearly 36 months after initiating RT and achieved a favorable radiographic response in all treated lesions.

**Conclusion:**

This case report demonstrates that dose-escalated MRgRT can treat anatomically unfavorable pancreatic SPT and achieve favorable long-term efficacy without significant toxicity. These data add to the growing body of evidence that MRgRT may significantly improve the therapeutic ratio over computed tomography (CT)–guided RT for complex abdominal tumors by facilitating tumor dose escalation while meeting OAR constraints.

## Introduction

Solid pseudopapillary tumors (SPTs) are rare epithelial neoplasms of the pancreas with an unknown origin and unclear pathogenesis, accounting for ~2% of all exocrine pancreatic tumors (1). First described by Frantz et al. in 1959 (2), SPTs are most common in females in their 20s–40s and are most frequently located in the tail of the pancreas (1, 3, 4). They are classified as low-grade malignant tumors, characterized by loosely cohesive, uniform epithelial cells forming both solid and pseudopapillary structures (5). SPTs typically grow slowly and rarely metastasize (1, 3, 4, 6).

Patients with SPTs are typically asymptomatic until the tumor reaches a considerable size, at which point they may compress adjacent organs, leading to abdominal pain, nausea, anorexia, weight loss, and/or a palpable abdominal mass (7). In rare cases, patients may experience jaundice or obstruction of the main pancreatic duct (3, 4, 8).

While outcomes for resectable SPTs are excellent, with 5-year overall survival (OS) of ~95% (9), unresectable disease carries a poorer prognosis with 5-year OS of ~50% (10). For advanced SPTs, systemic therapies, including cytotoxic chemotherapy (11–14), mTOR inhibitors (15, 16), and anti-angiogenic tyrosine kinase inhibitors (17), have been reported in small series, but clinical benefit is uncertain and long-term local control (LC) is uncommon.

Radiotherapy (RT) may improve LC and symptom relief versus chemotherapy alone for pancreatic SPT (18, 19). While the optimal RT dose is uncertain, a higher biologically effective dose (BED) may provide more substantial and durable local efficacy, as has been demonstrated for pancreatic ductal adenocarcinoma (20–22). The only published outcomes of ablative RT for pancreatic SPT are from a case report of proton therapy prescribed at 67.5 GyE in 25 fractions (BED_10_ = 85.73 Gy_10_) to a lymph node recurrence after pancreaticoduodenectomy that resulted in durable LC (23).

Herein we reported the first clinical outcomes, to the best of our knowledge, of ablative magnetic resonance–guided radiotherapy (MRgRT) for advanced pancreatic SPT.

## Case presentation

**Figure 1:**
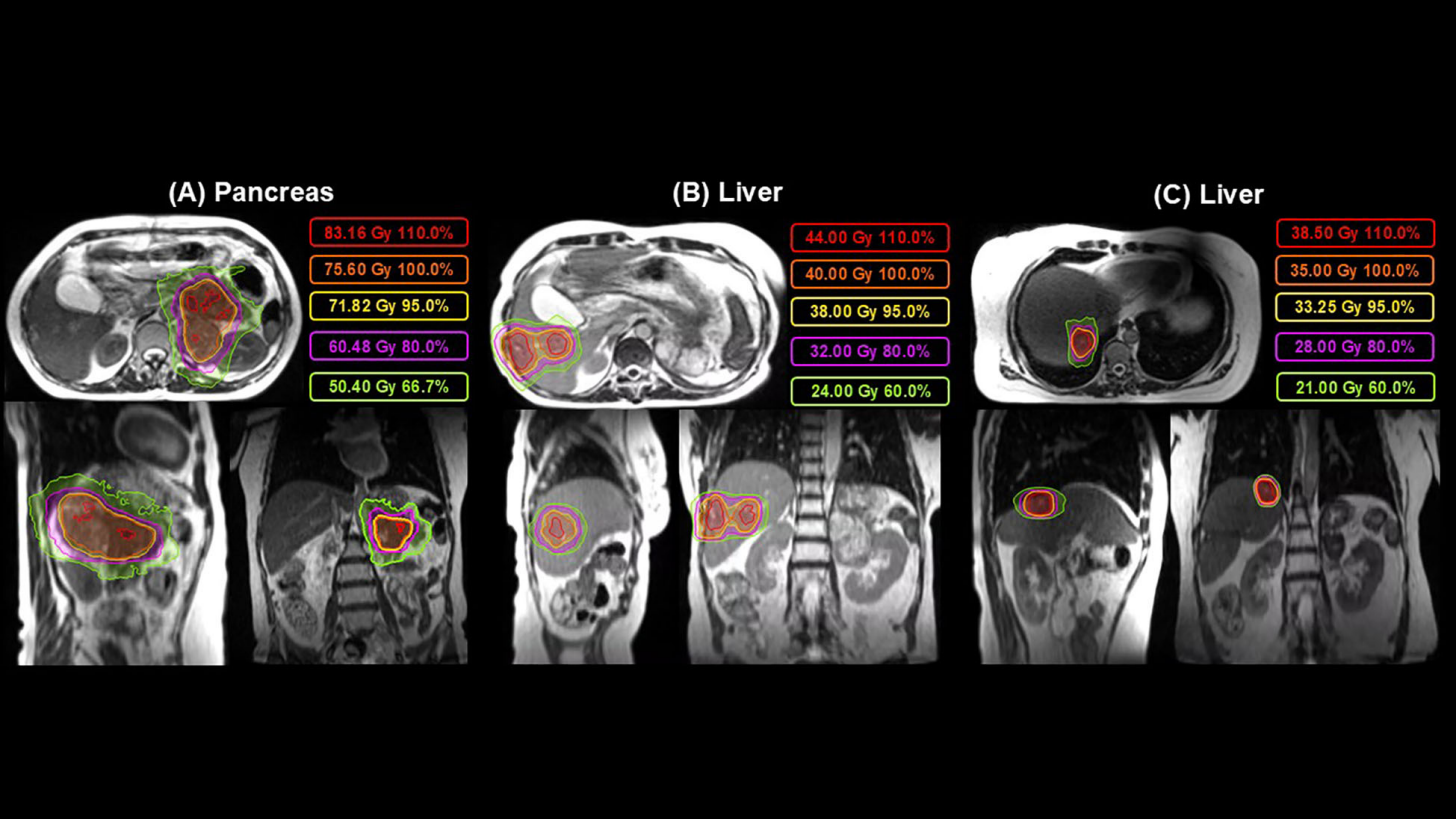
Radiotherapy treatment planning for a **(A)** solid pseudopapillary tumor of the pancreas prescribed with a dose-painting technique to a dose of 75.6 Gy to the central tumor and 50.4 Gy periphery in 28 fractions in October 2022, **(B)** two liver metastasis prescribed to a dose of 40.0 Gy in 5 fractions in December 2022, and **(C)** one liver metastasis prescribed to a dose of 35 Gy in 1 fraction in October 2024.

A 63-year-old Hispanic female with no significant past medical history underwent a workup for fatty liver, including an abdominal ultrasound that incidentally detected a mass in the liver. Further workup, including contrast-enhanced abdominal computed tomography (CT) scans, revealed 2 enhancing lesions in hepatic segment 6 measuring 2.8 cm × 1.0 cm and 1.1 cm × 0.9 cm, respectively. Additionally, a lobulated, ill-defined 7.0 cm × 5.0 cm soft-tissue mass was identified involving the pancreatic body with no vascular encasement. Liver biopsy confirmed a diagnosis of SPT of the pancreas. A contrast-enhanced MRI scan of the abdomen using the gadolinium-based contrast agent gadoteridol (**Figure 1B**) shortly thereafter demonstrated that the pancreatic tail mass measured 10.0 cm × 8.0 cm × 8.7 cm and was inseparable from the stomach, small intestine, left adrenal gland, left kidney, and the diaphragmatic crura and encased the splenic vein and artery and abutted the left renal vein and artery. The MRI scan also showed the two previously seen hepatic segment 6 metastases, but also a sub-centimeter segment 7 metastasis. Routine laboratory tests, including carcinoembryonic antigen (CEA) and CA 19-9, were within normal limits.

There was consensus from our multidisciplinary tumor board that the patient should receive chemotherapy followed possibly by surgery depending on treatment response. The patient was originally planned for four cycles of gemcitabine/cisplatin, although after the first cycle she developed ototoxicity, leading to the discontinuation of cisplatin. She ultimately received one cycle of cisplatin and completed all four planned cycles of gemcitabine.

Restaging MRI after chemotherapy demonstrated stable disease, and the patient was subsequently taken to surgery for planned resection. Intraoperatively, however, the tumor was found to be more locally advanced than radiographically apparent, with encasement of the superior mesenteric portal vein confluence, greater than expected abutment of the superior mesenteric artery, invasion of the jejunum and duodenum, and extension into the paravertebral space. The tumor was deemed unresectable, and the procedure was aborted.

The patient completed four cycles of FOLFOX, and because there was no evidence of progression, she was referred for definitive RT. Our department offers multiple advanced RT platforms, including both photon and proton therapies, and because of the large size and unfavorable anatomic location of the primary tumor abutting gastrointestinal (GI) organs at risk (OARs), we felt that MRgRT using a 0.35-Tesla MR-Linac would offer the most favorable therapeutic ratio.

Treatment was delivered in the supine position with continuous cine-MRI throughout treatment, automatic beam gating, and a mid-inspiration breath-hold technique. The gross tumor volume (GTV) was defined as the visible tumor on the simulation and diagnostic imaging scans. A clinical target volume (CTV) was not used, and because the MR-Linac treats with automatic beam gating, an integral target volume (ITV) to account for motion was not used. The planning target volume (PTV) margin was created from an isotropic 3 mm expansion of the GTV. The primary tumor was prescribed to 50.4 Gy in 28 fractions with a dose-painted simultaneous integrated boost to 75.6 Gy (BED_10_ = 96.6 Gy_10_) in 28 fractions. The boost volume was created from a 1-cm contraction of the GTV. Dose constraints to target volumes and OARs are summarized in **Table 1**. For each fraction, the daily MRI was reviewed to confirm that the target and surrounding OARs were consistent with the reference plan. If an anatomic change raised concern for target coverage or a possible OAR constraint violation, adaptive recontouring and predicted dose evaluation were performed before treatment delivery. No treatment was delivered under conditions that required further plan modification. All reported dose evaluation metrics were derived from the reference treatment plan. The GTV volume was 276.54 cc. The mean GTV dose was 68.83 Gy, and the dose to 95% (D95) and 90% (D90) of the GTV volume were 53.07 Gy and 54.36 Gy, respectively. The 75.6 Gy boost PTV had a volume of 55.08 cc, received a mean dose of 81.52 Gy, and had D95 and D90 values of 78.80 Gy and 79.48 Gy, respectively.

**Table 1:** Summary of dose constraints used during treatment planning for (**A**) pancreas, (**B**) two liver lesions in segment VI, and (**C**) liver lesion in segment VII.

A. Pancreas treatment (75.6 Gy in 28 fractions)	
Structure	Constraint
PTV50.4	≥ 95.0% at 50.4 Gy
GTV	≥ 99.0% at 50.4 Gy
Stomach	≤ 0.03 cc at 54.0 Gy
Large bowel	≤ 0.03 cc at 54.0 Gy
Small bowel	≤ 0.03 cc at 54.0 Gy
Duodenum	≤ 0.03 cc at 54.0 Gy
Spinal canal	≤ 0.03 cc at 54.0 Gy
Kidneys	Mean ≤ 18.0 Gy
Liver	Mean ≤ 18.0 Gy
B. Segment VI liver metastases (40.0 Gy in 5 fractions)	
Structure	Constraint
GTV_Lower	≥99.0% at 40.0 Gy
Small bowel	≤ 0.50 cc at 35.0 Gy; ≤ 0.03 cc at 40.0 Gy
Stomach	≤ 0.50 cc at 35.0 Gy; ≤ 0.03 cc at 40.0 Gy
Large bowel	≤ 0.50 cc at 38.0 Gy; ≤ 0.03 cc at 43.0 Gy
Spinal canal	≤ 0.03 cc at 20.0 Gy
Kidneys	Mean ≤ 4.0 Gy
Liver-GTV	Mean ≤ 10.0 Gy; ≤ 700.0 cc at 21.0 Gy
Chest wall	≤ 30.0 cc at 30.0 Gy
C. Segment VII liver metastasis (35.0 Gy in 1 fractions)	
Structure	Constraint
GTV	≥ 99.0% at 35.0 Gy
CTV	≥ 99.0% at 35.0 Gy
Liver-GTV	Mean ≤ 6.6 Gy; ≤ 700.0 cc at 9.10 Gy
Spinal canal	≤ 0.03 cc at 6.0 Gy
Skin_5mm	≤ 10.00 cc at 23.0 Gy; ≤ 0.03 cc at 26.0 Gy
Chest Wall	≤ 1.00 cc at 22.0 Gy; ≤ 5.00 cc at 27.0 Gy; ≤ 0.03 cc at 30.0 Gy
Esophagus	≤ 10.00 cc at 11.2 Gy; ≤ 0.03 cc at 12.4 Gy
Stomach + Duodenum	≤ 10.00 cc at 9.0 Gy; ≤ 5.00 cc at 11.2 Gy; ≤ 0.03 cc at 12.4 Gy
Small bowel	≤ 10.00 cc at 9.0 Gy; ≤ 5.00 cc at 11.2 Gy; ≤ 0.03 cc at 12.4 Gy
Large bowel	≤ 10.00 cc at 9.0 Gy; ≤ 5.00 cc at 11.2 Gy; ≤ 0.03 cc at 18.4 Gy
Kidney	Mean ≤ 7.0 Gy; ≤ 33.0% at 10.0 Gy
Heart	≤ 15.00 cc at 16.0 Gy; ≤ 0.03 cc at 22.0 Gy
Chest wall	≤ 28.0 cc at 11.0 Gy; ≤ 2.00 cc at 17.0 Gy

PTV, planning target volume; GTV, gross tumor volume; CTV, clinical target volume; Liver-GTV, liver volume excluding the GTV.

She tolerated RT to the primary tumor very well and did not experience grade 2 or higher toxicity at any time. One month later, we treated two of the three liver metastases concurrently with the MR-Linac to 40 Gy in 5 fractions (BED_10_ = 72.0 Gy_10_). The hepatic lesion in segment 7 was not treated at the time out of concern regarding cumulative liver dose and its small size, and our plan was to consider treating it if there was future isolated progression. She did not experience grade 2 or higher toxicity at any time related to RT for the liver metastases.

**Figure 2:**
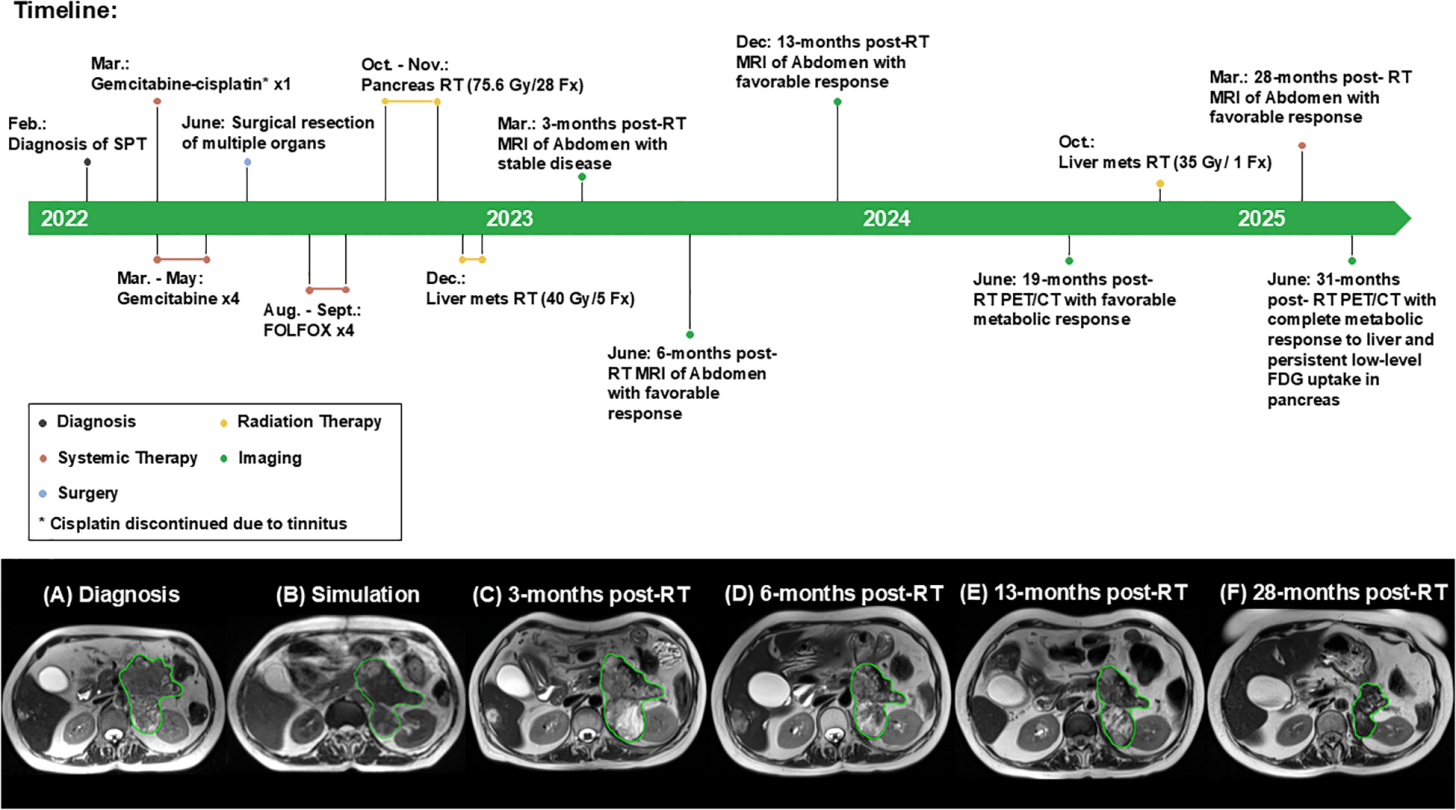
Summary of the patient’s clinical course. Timeline outlining major clinical events following the diagnosis of a solid pseudopapillary tumor of the pancreas. **(A–F)** Serial abdominal MRI scans demonstrating disease status and response to pancreas radiation therapy over time. Abbreviations: SPT, solid pseudopapillary tumor; RT, radiation therapy.

The patient was then followed with restaging imaging and clinical evaluation every 3 to 6 months (**Figure 1**). Restaging scans showed a gradual size reduction in all treated lesions without progression elsewhere. Nineteen months after completing dose-escalated MRgRT, a positron emission tomography (PET)/CT scan demonstrated continued reduction in the primary tumor size (now 8.4 cm × 4.6 cm) and declining metabolic activity of the pancreatic lesion [standard uptake value (SUV) max of 2.9], although the untreated segment 7 hepatic lesion had increased metabolic uptake and slight enlargement for the first time. An MRI scan confirmed that this lesion now measured 1.6 cm × 1.1 cm, and it was treated with a prescribed dose of 35 Gy in one fraction (BED_10_ = 157.5 Gy_10_). An overview of all RT treatment plans delivered is provided in **Figure 2**.

At the time of last follow-up 35.5 months after initiation of RT to the primary pancreatic tumor, the patient remains alive, is asymptomatic with excellent quality of life, and has no evidence of progressive disease. The pancreatic lesion continues to slowly decrease in size, most recently measuring 7.7 cm × 4.0 cm with minimal fluorodeoxyglucose (FDG) avidity (SUV max of 2.5) and no signs of disease progression. She did not experience more than grade 1 toxicity related to all courses of RT. CEA and CA 19–9 have remained within normal limits since the completion of MRgRT.

## Discussion

SPTs of the pancreas are rare, low-grade malignant neoplasms that typically have favorable outcomes when surgical resection is possible, although outcomes are significantly poorer for patients with unresectable disease. Surgical resection is the definitive treatment, offering curative potential even in cases with local invasion or limited metastasis, as longer-term recurrence-free survival is common post-resection (24). However, surgical resection may be limited by medical comorbidities, tumor location, or locally advanced disease, highlighting the need for a personalized, multidisciplinary treatment approach.

Systemic therapy has been reported in only small series, with no established regimen and uncertain benefit in unresectable, recurrent, or metastatic SPTs (11, 13). As a result, local treatment options such as RT merit consideration, particularly when durable control of an unresectable primary tumor or isolated metastasis may meaningfully affect clinical outcomes and quality of life. This unmet need for safe and effective nonoperative alternatives provides the rationale for exploring advanced image-guided RT techniques, including MRgRT, in the management of select patients with SPTs.

RT for SPTs has been described only in a small number of case reports (**Table 2**). Because RT has been used in many different clinical settings, we included all published cases in which RT was delivered, whether given alone or alongside surgery or systemic therapy. In nearly all reports, RT was delivered with conventional photon techniques, typically 27–54 Gy, and used mainly for symptom relief or disease stabilization rather than definitive ablation (18, 23, 25–29). Notably, Kapoor et al. reported a postoperative case treated with 50 Gy in 25 fractions that achieved a complete remission lasting 12 years, demonstrating that conventional RT can still provide durable LC in selected high-risk patients (28).

**Table 2:** Summary of published cases describing the use of radiotherapy for solid pseudopapillary tumors of the pancreas.

Reference	Country	Age/sex	Pancreas site + extent of disease	Tumor size (cm)	Previous treatment	Modality	Dose fractionation schedule (BED)	Overall survival
Fried et al., 1985[25]	USA	18/F	Head + Portal vein, hepatic artery	NR	None	Photon	40 Gy/20 fx (BED_10_: 48 Gy_10_)	3 years
Cappellari et al., 1990[26]	USA	32/F	Head + SMV, Liver	15 × 20	None	Photon	Liver: 30 Gy/15 fx (BED_10_: 36 Gy_10_) Pancreas:30 Gy/15 fx + Boost 10 Gy/5 fx (BED_10_: 48 Gy_10_)	> 2.5 years
Maffuz et al., 2005 [11]	Mexico	23/F	Head + Mesocolon, porta hepatis, gastrocolic ligament	13	None	Photon	50.4 Gy/28 fx (BED_10_: 59.5 Gy_10_)	NR
Zauls et al., 2006[18]	USA	33/F	Body + PSC, IMV, SMV	6.3 × 6.5	None	Photon	50.4 Gy/28 fx (BED_10_: 59.5 Gy_10_)	18 months
Wojciak et al., 2018[27]	Poland	51/F	Body + Liver, LN	NR	Surgery, Chemo, LT	Photon	27 Gy/15 fx (BED_10_: 31.9 Gy_10_)	1.5 years
Kapoor et al., 2019[28]	India	45/F	Tail + Capsular Invasion	7 × 7 × 5	Surgery	Photon	50 Gy/25 fx (BED_10_: 60.0 Gy_10_)	12 years
Kodama et al., 2020[23]	Japan	50s/M	Head + Liver, LN	7 × 4 × 6.5	Surgery, Chemo	Proton	67.5 GyE/25 fx (BED_10_: 85.7 Gy_10_)	3 years
Rajahraman et al., 2024[29]	USA	77/F	Operative bed + Gastric Fundus	4 × 3.5 × 4.4	Surgery, Chemo	Photon	54 Gy/27 fx + Boost: 18Gy /6 fx (BED_10_: 88.2 Gy_10_)	~ 4 months
Current study	USA	63/F	Body + Liver	10.0 × 8.0 × 8.7	Chemo	Photon	Pancreas:75.6 Gy/28 fx (BED: 96.6 Gy_10_) Liver: 40 Gy/5 fx (BED: 72.0 Gy_10_) Liver: 35 Gy/1 fx (BED: 157.5 Gy_10_)	35.5 months

ST, Systemic Therapy; F, Female; M, Male; Fx, Fraction; PSC, portosplenic confluence; IMV, inferior mesenteric vessels; SMV, superior mesenteric vessels; LT, liver transplant, LN, Lymph Nodes; BED, biological effectiveness dose.

To date, the only published outcomes of ablative RT are from a case report of proton therapy for a nodal recurrence after surgery by Kodama et al. (23). Although radiation dose escalation may improve the probability of long-term LC, it may not be feasible, especially for anatomically unfavorable tumors using CT-guided RT that is limited by suboptimal image quality, lack of intrafraction volumetric imaging, and an inability to rapidly adapt treatment to account for interfraction anatomic change. MRgRT offers solutions to these challenges and enables safe dose escalation, a strategy increasingly adopted in modern practice (30). At our institution, MRgRT utilization has grown substantially in recent years, especially for pancreatic and other mobile abdominal tumors, with a clear shift toward ultra-hypofractionated and ablative regimens (31). The ability to deliver higher doses in fewer fractions, including single-fraction treatment in selected settings as shown in our phase 2 SMART ONE trial (32), and further supported by a recently published multi-institutional analysis of MR-guided single-fraction ablative RT demonstrating favorable feasibility, safety, and efficacy outcomes (33), supports the expectation that MRgRT may improve long-term clinical outcomes in appropriately selected patients. This rationale informed our decision to use dose-escalated MRgRT for both the patient’s large primary pancreatic tumor and oligometastatic liver disease with the goal of achieving durable LC, which may not have been achieved if using a standard CT-guided linac without online adaptive capabilities.

Our case highlights the promising potential of MRgRT for the management of unresectable pancreatic SPTs. Delivering dose-escalated treatment on an MR-Linac resulted in substantial tumor regression, durable control of the pancreatic primary, and complete response in all treated liver metastases, while preserving excellent quality of life and causing no acute or late toxicities. The patient has experienced sustained clinical and radiographic benefit nearly 3 years after treatment. The ability to visualize soft-tissue anatomy throughout RT provided the confidence needed to safely escalate dose in a tumor abutting critical organs, an approach that would have been difficult using conventional CT-guided techniques. While broader experience and longer follow-up are needed, these findings suggest that MRgRT may offer a precise, well-tolerated, and potentially definitive local therapy for select patients with SPTs who are not surgical candidates. As MRgRT techniques continue to evolve, this case may inform the management of similar presentations and supports consideration of dose-escalated MRgRT within multidisciplinary care for this rare disease.

## Data Availability

The datasets presented in this article are not readily available because this is a case report and due to legal and ethical issues, patient level data used are unavailable (medical confidentiality). Requests to access the datasets should be directed to the corresponding author: Michael Chuong, MichaelChu@Baptisthealth.net.
